# Changes in Mouse Thymus and Spleen after Return from the STS-135 Mission in Space

**DOI:** 10.1371/journal.pone.0075097

**Published:** 2013-09-19

**Authors:** Daila S. Gridley, Xiao Wen Mao, Louis S. Stodieck, Virginia L. Ferguson, Ted A. Bateman, Maria Moldovan, Christopher E. Cunningham, Tamako A. Jones, Jerry M. Slater, Michael J. Pecaut

**Affiliations:** 1 Department of Basic Sciences, Division of Radiation Research, Loma Linda University, Loma Linda, California, United States of America; 2 BioServe Space Technologies, Aerospace Engineering Sciences, University of Colorado, Boulder, Colorado, United States of America; 3 Department of Mechanical Engineering, University of Colorado, Boulder, Colorado, United States of America; 4 Department of Bioengineering, University of North Carolina, Chapel Hill, North Carolina, United States of America; Texas Tech University, United States of America

## Abstract

Our previous results with flight (FLT) mice showed abnormalities in thymuses and spleens that have potential to compromise immune defense mechanisms. In this study, the organs were further evaluated in C57BL/6 mice after Space Shuttle Atlantis returned from a 13-day mission. Thymuses and spleens were harvested from FLT mice and ground controls housed in similar animal enclosure modules (AEM). Organ and body mass, DNA fragmentation and expression of genes related to T cells and cancer were determined. Although significance was not obtained for thymus mass, DNA fragmentation was greater in the FLT group (P<0.01). Spleen mass alone and relative to body mass was significantly decreased in FLT mice (P<0.05). In FLT thymuses, 6/84 T cell-related genes were affected versus the AEM control group (P<0.05; up: *IL10*, *Il18bp*, *Il18r1*, *Spp1*; down: *Ccl7*, *IL6*); 15/84 cancer-related genes had altered expression (P<0.05; up: *Casp8, FGFR2, Figf, Hgf*, *IGF1*, *Itga4, Ncam1, Pdgfa, Pik3r1, Serpinb2*, *Sykb*; down: *Cdc25a*, *E2F1, Mmp9, Myc*). In the spleen, 8/84 cancer-related genes were affected in FLT mice compared to AEM controls (P<0.05; up: *Cdkn2a*; down: *Birc5*, *Casp8*, *Ctnnb1*, *Map2k1*, *Mdm2*, *NFkB1, Pdgfa*). Pathway analysis (apoptosis signaling and checkpoint regulation) was used to map relationships among the cancer–related genes. The results showed that a relatively short mission in space had a significant impact on both organs. The findings also indicate that immune system aberrations due to stressors associated with space travel should be included when estimating risk for pathologies such as cancer and infection and in designing appropriate countermeasures. Although this was the historic last flight of NASA’s Space Shuttle Program, exploration of space will undoubtedly continue.

## Introduction

Astronaut health and safety are of key importance to the success of long-term missions in space. The immune system consists of a complex network of organs, tissues and cells that are essential for both maintenance of homeostasis in the body and for defense against infectious microbes and aberrant cell types with potential to progress to cancer. Additional pathologies associated with immune dysfunction include autoimmunity, hypersensitivity and poor wound healing. Although understanding of the underlying mechanisms remains limited, there are numerous reports that stressors in the space flight environment, e.g., microgravity, radiation and psychological stress of confinement, can have a profound impact on immune system status [1-16]. A better understanding of space flight-related health issues is needed, particularly when missions proceed beyond Earth’s protective geomagnetic field. Even more importantly, knowledge gained on the biological effects of factors in the space flight environment is relevant also to the general public as high altitude commercial airline flights and space tourism will undoubtedly continue to increase.

Current predictions of health consequences for space flight personnel, including risk for cancer, remain based on minimal data with large uncertainties [17,18]. If flight-related stressors result in higher mutation rates, as has been reported for bacteria [19], and/or modulated gene expression patterns [20,21], the possibility for malignant transformation could certainly be increased. This possibility together with radiation-induced immune depression or dysfunction due to a solar particle event (SPE) and reactivation of endogenous viruses with oncogenic potential, e.g. Epstein-Barr virus [22,23], could further increase the risk for cancer. In addition, it has been recently reported that microgravity was the cause for impaired human T cell activation during space flight due to inhibition of immediate early gene transcription [24].

Induction of thymic lymphoma in mice is a classical model for studying radiation-induced carcinogenesis [25]. In addition, the thymus serves as a source of T cell reconstitution after great loss of this leukocyte type [26], which could certainly result from radiation exposure during an SPE. Our ground-based mouse studies using space-relevant radiation, including simulated SPE, have shown dramatic loss of leukocytes and effects on other parameters in various body compartments [27-34]. Proliferation of sub-lethally damaged cells during reconstitution, regardless of specific site of origin, increases the risk for transformation to a malignant phenotype. In addition, dysregulation of T cell maturation would have a negative impact on cell functions and interactions after their transit to other body sites, thus compromising capacity to destroy malignant or potentially malignant cells throughout the body. Our interest in the spleen comes from the fact that it is a major source of immune cells that function continuously via humoral and cell-mediated pathways. It is also a major site at which antigen presentation and production of opsonins that facilitate phagocytosis of bacteria and other particulate materials occurs.

We have previously evaluated thymus, spleen and other tissue responses obtained from mice flown to and from the International Space Station (ISS) onboard Space Shuttle Endeavour, i.e. Space Transportation System 77 (STS-77), STS-108 and STS-118 [20,35-42]. Numerous differences were noted in the flight (FLT) mice compared to control animals on ground. An intriguing finding especially relevant to the present study was that the expression of 30/84 genes related to cancer was significantly modulated in the thymus from FLT mice flown on STS-118 compared to ground controls housed in similar animal enclosure modules (AEM).

For the present study, thymus and spleen samples were collected from mice on July 21, 2011 at the Kennedy Space Center (KSC) after a 13-day flight of Space Shuttle Atlantis (STS-135). This was the historic final flight of NASA’s Space Shuttle Program. Our overall hypothesis was that important organs of the immune system in FLT mice would have excessive DNA fragmentation and altered expression of genes related to T helper (Th) cells and cancer. This study was part of the NASA Ames Research Center’s Biospecimen Sharing Program of Commercial Biomedical Test Module-3 (CBTM-3) and collaboration among investigators at the NASA Ames Research Center, BioServe Space Technologies and Amgen, Inc.

## Materials and Methods

### Animals and Housing

Growing (9 weeks old at start of experiment) female C57BL/6 mice (Charles River Laboratories, Inc., Wilmington, MA) were used in this study. FLT mice were housed in AEM, enclosed habitats with a raised mesh floor that provides ventilation, lighting and waste collection, as well as food and water for the rodents. The ground controls were also housed in flight hardware (AEM group). The environment (e.g., temperature, humidity, CO_2_ level) for the AEM mice was kept equivalent to that of the FLT mice based on 48 h delayed telemetry data. For example, temperature was 26-28°C and a 12-h light:dark cycle was maintained. Both groups of animals were fed a reformulated rodent food bar diet approved by NASA. Consumption of food and water was monitored daily throughout this study.

The LLU IACUC was consulted for our portion of the study and no protocol was required, since only tissues obtained after euthanasia (no live animals) were processed at our institution. All NASA activities involving vertebrate animals are carried out in strict accordance with the recommendations in the Guide for the Care and Use of Laboratory Animals of the National Institutes of Health (NIH). The animal studies performed were all approved by multiple ACUC review boards in accordance with NASA’s standard operating procedures including the NASA Ames Research Center ACUC, the NASA, Kennedy Space Center ACUC, the University of Colorado at Boulder IACUC (this is the home institution for the Principal Investigator of the entire study – Dr. V. L. Ferguson - where tissues were collected for analysis as presented in this paper). These three ACUCs reviewed and approved all of our protocols as listed above: "in strict accordance with the recommendations in the Guide for the Care and Use of Laboratory Animals of the NIH.”

### Sample Collection

All dissections occurred at the Space Life Sciences Laboratory (SLSL) at KSC within 3-5 h after return of the Space Shuttle Atlantis from a 13-day mission to the ISS. Mice, approximately 11 weeks of age, were euthanized using 4% isoflurane followed by cardiac puncture and exsanguination. Tissues were distributed across a large team of investigators under the guidance/organization of the NASA’s Biospecimen Sharing Program. Thymuses and spleens were extracted and prepared in 4% paraformaldehyde (thymus) or snap frozen in liquid nitrogen (thymus and spleen). The number of samples shipped overnight to Loma Linda University (LLU) for analyses were as follows: thymus (AEM n = 7, FLT n = 4) and spleen (AEM n = 8, FLT n = 5).

### Thymus and Spleen Mass, Alone and Relative to Body Mass (RTM, RSM)

Mice were first weighed by personnel at the SLSL shortly after the Space Shuttle landed. The animals were then euthanized and their thymuses and spleens were excised and weighed shortly thereafter. The following formula was used to calculate organ mass relative to body mass: RTM (or RSM) = organ mass (mg)/body mass (g). Means for thymus mass and RTM represent 7 AEM and 4 FLT mice; for spleen mass and RSM, values represent 8 AEM and 5 FLT mice.

### TUNEL Assay on Thymus

The thymus was evaluated using the terminal deoxynucleotidyl transferase dUTP nick end labeling (TUNEL) assay according to standard procedures. Data were obtained for 6 AEM and 3 FLT mice. The assay detects DNA fragmentation by incorporating fluorescein-12-dUTP at 3’-OH DNA ends using the recombinant terminal deoxynucleotidyl transferase enzyme (rTdT). Briefly, 6 µm paraffin embedded thymus sections were processed using an *in situ* cell death detection kit (DeadEnd Fluorometric TUNEL system, catalog no. TB235; Promega, Madison, WI) according to the manufacturer’s instructions. After deparaffinization, rehydration, fixation and permeabilization steps, slides were incubated with a mixture of fluorescein-labeled nucleotides and rTdT at 37°C for 1 h. rTdT catalyzes the incorporation of fluorescein nucleotides to free 3’-OH terminals of DNA fragments. Slides incubated with fluorescein-labeled nucleotide mixture without rTdT served as negative control. Cells treated with DNAse I (10 units/ml; Sigma Aldrich, St. Louis, MO) to induce breaks in DNA strands served as positive control. Fluorescence microscopy was performed with a microscope (BX61; Olympus, Central Valley, PA). For quantitative analysis, numbers of apoptotic cells were counted in nine sections from each animal. The surface of each section was measured on digital microphotographs using ImageJ v. 1.4 (available as freeware from http://rsbweb.nih.gov/ij/). Density profiles were expressed as mean number of apoptotic cells per square millimeter. Similar procedures have been described in the literature [43,44].

### Gene Expression in Thymus and Spleen

Frozen thymus and spleen samples were thawed before quantitative reverse transcriptase-polymerase chain reaction (RT-PCR) analysis. For the thymus, expression of 84 genes using the Mouse Th1-Th2-Th3 RT^2^ Profiler^TM^ PCR Array (PAMM-034A) and 84 genes using the Mouse Cancer PathwayFinder RT^2^ Profiler^TM^ PCR Array (PAMM-033A) was determined. For genes in the spleen, the same cancer array was used as for the thymus. Both arrays were obtained from SABiosciences/Qiagen Corp., Frederick, MD and the RT-PCR was performed at the SABiosciences Technical Core. The details of the procedures have been previously reported [27]. Briefly, the extracted RNA was run on a bioanalyzer (Agilent Technologies, Santa Ana, CA) and its integrity was confirmed by assessing 18s and 28s rRNA peaks and by RNA integrity number (RIN). Spectrophotometrical measurements showed that 260/280 and 260/230 ratios for all samples were above 2.0 and 1.7, respectively. PCR reactions were performed on a Biorad cycler (Bio-Rad Laboratories, Hercules, CA) using RT^2^ Real-Time^TM^ SYBR Green PCR Master Mix PA-011 (SABiosciences/Qiagen) and relative changes were calculated using the C_t_ (threshold cycle) method; five housekeeping genes, RT controls and positive PCR controls were included. Comparison was made between the FLT versus AEM ground controls.

### Pathway Analysis

Ingenuity Pathway Analysis (IPA) (Ingenuity® Systems, Redwood City, CA; www.ingenuity.com) was used to map some of the more important relationships among the characterized cancer-related genes. The assessments included both the thymus and spleen. Due to the nature of the genes assessed, the limited number of significant changes and the tissue sources, we relaxed the statistical constraints for the pathway analysis and focused on two specific canonical pathways: Myc Mediated Apoptosis Signaling and Cell Cycle: G1/S Checkpoint Regulation. Comprehensive description of the legend in the IPA figures can be found here: http://ingenuity.force.com/ipa/articles/Feature_Description/Legend.

### Statistical Analysis

Gene expression data were analyzed using Student’s *t*-test at the SABiosciences/Qiagen Technical Core. This statistical test is widely accepted as valid when used for results after relative quantification with RT-PCR, as was done in the present study. Other data were analyzed using Student’s T test (Sigmaplot 12.3; Systat Software, Inc., Chicago, IL). Means and standard error of means (SEM) are reported. *P* values less than 0.05 were selected to indicate significance.

## Results

### Food and Water Consumption

Intake of food and water was monitored daily. Food consumed per mouse per day was virtually identical for the AEM and FLT groups, i.e., 4.08 g and 4.09 g, respectively. Water consumption, however, between the two groups was somewhat different: 3.38 ml/mouse/day for the AEM group and 2.73 ml/mouse/day for the FLT group.

### Thymus and Spleen Mass Alone and Relative Organ Mass (RTM and RSM)

**Figure 1:**
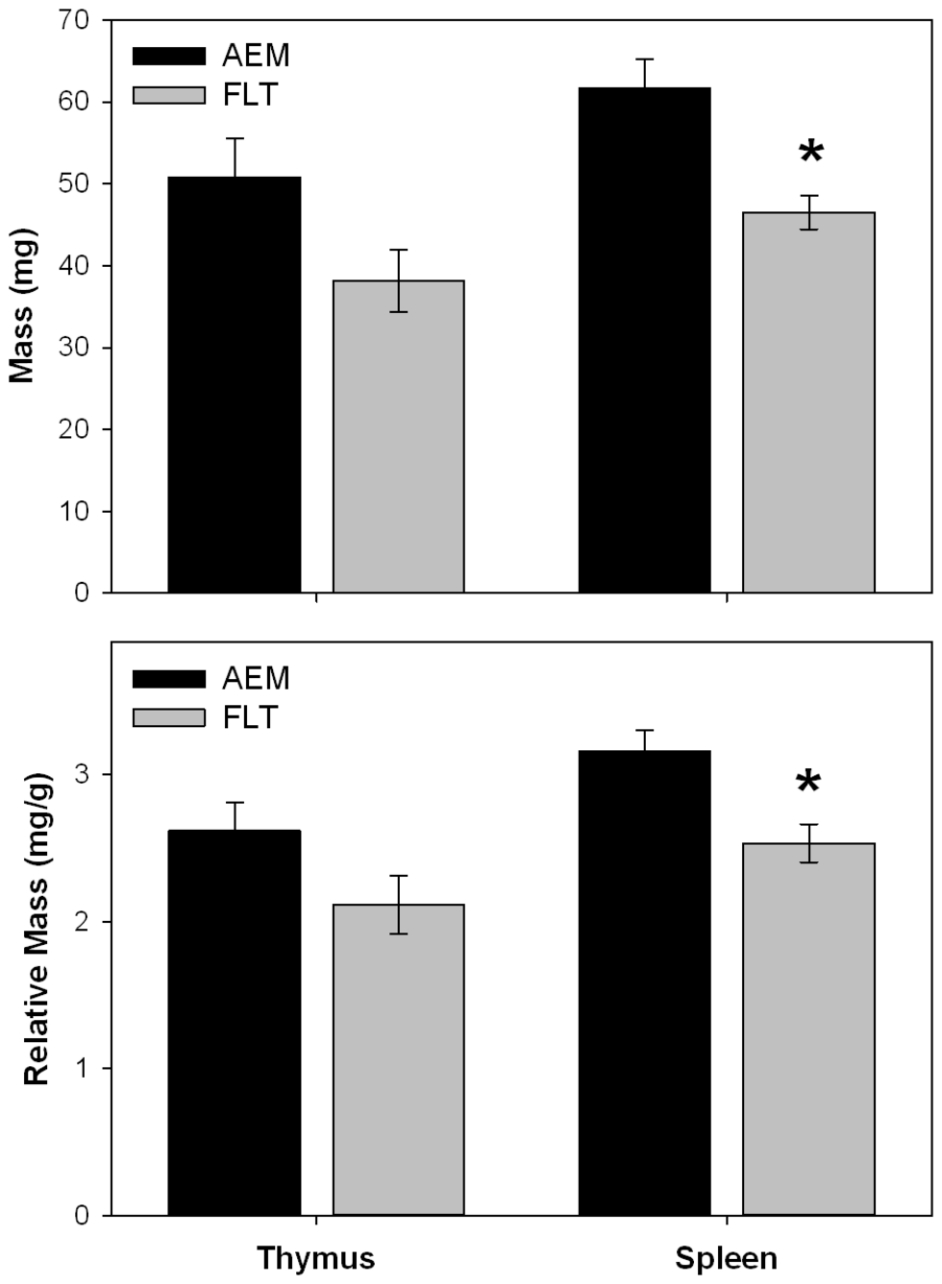
Thymus and spleen mass alone and relative to body mass (RTM, RSM). Bars represent mean ± SEM. For thymus mass and RTM, n = 7 (AEM group) and n = 4 (FLT group). For spleen mass and RSM, n = 8 (AEM group) and n = 5 (FLT group). AEM, animal enclosure module (control mice housed on ground); FLT, flight mice. *P<0.05 vs. AEM. For FLT vs. AEM thymus mass, P=0.101.

Prior to take-off, body mass was very similar for the two groups; the FLT and AEM mice weighed 20.3 ± 1.2 g and 20.7 ± 1.2 g, respectively. Body mass shortly after landing was also not significantly different between the two groups: 18.1 ± 0.5 g (FLT) and 19.3 ± 0.5 g (AEM). The mass of both organs alone and relative to body mass are shown in Figure 1. Although the thymus mass and RTM values were relatively low for the FLT group compared to the AEM group, statistical significance was not obtained possibly due to low sample size. For the FLT group, the decrease in thymus mass alone approached significance (P=0.101 vs. AEM). However, Figure 1 also shows that the FLT animals had significantly lower spleen mass and RSM values compared to the AEM ground controls (P<0.05).

### DNA Fragmentation in Thymus

**Figure 2:**
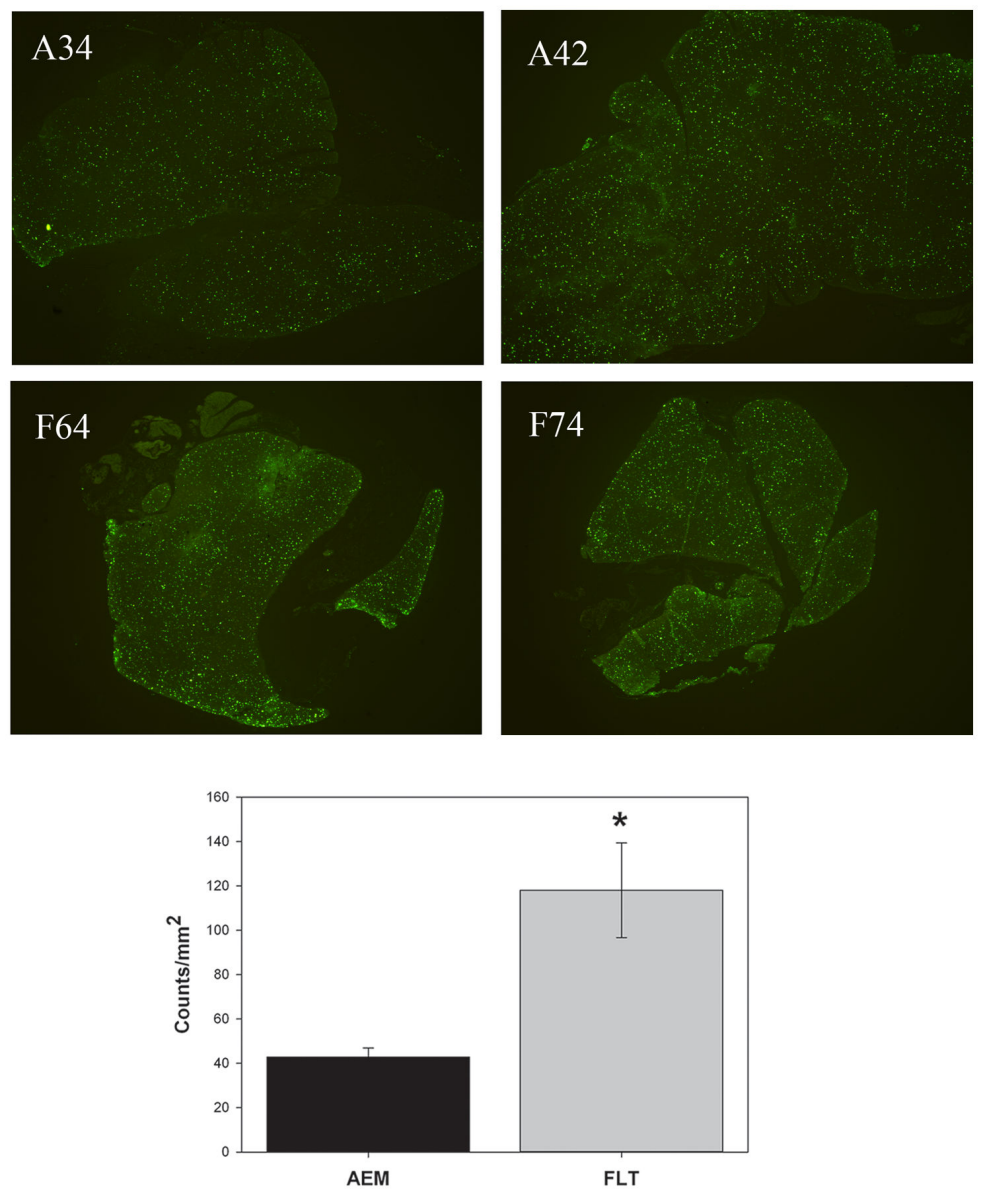
DNA fragmentation in thymus. The terminal deoxynucleotidyl transferase dUTP nick end labeling (TUNEL) assay was used. The top panel shows two representative examples from each of the groups. AEM (A34 and A42): control mice housed on ground in animal enclosure modules; FLT (F64 and F74): flight mice. The bottom panel shows the results after quantification; bars represent mean ± SEM for 6 AEM and 3 FLT mice. *P<0.01 vs. AEM.

Representative images of DNA fragmentation in thymuses obtained from two mice in each of the groups are presented in the top four panels of Figure 2. Quantification of fluorescence, based on the TUNEL assay, indicated that there was a greater impact on the mice flown in space (Figure 2, bottom panel). The density of TUNEL-positive cells was significantly higher for FLT group compared to the AEM controls (P<0.01).

### Th Cell Subset-related Genes in Thymus

Genes related to the T cells with significant expression differences in FLT thymuses compared to the AEM counterparts housed on ground (P<0.05) are shown in Table 1. Of the 6 genes that were affected in the FLT group out of a total 84 evaluated, four were up-regulated (*Il10*, *Spp1*, *Il18r1*, *Il18bp*) and two were down-regulated (*Il6*, *Ccl7*). Fold-change was greater than 2.0 for all of these genes except *Il18bp*. Of the remaining genes, the expression of only *Il15* approached statistical significance (fold-change was 1.5; P<0.1 vs. AEM group).

**Table 1:** Fold-change in genes associated with Th cell subsets in the thymuses from FLT vs. AEM control group (P<0.05).

**Genes**	**Fold-change**	**Description**
Il10	2.97	Interleukin 10
Spp1	2.77	Secreted phosphoprotein 1; also known as osteopontin
Il18r1	2.01	Interleukin 18 receptor 1
Il18bp	1.75	Interleukin 18 binding protein
Il6	-2.71	Interleukin 6
Ccl7	-2.37	Chemokine (C-C motif) ligand 7

Data were obtained using the RT^2^ Profiler^TM^ PCR Array Mouse Th1-Th2-Th3 (PAMM-034A). There were 3 FLT and 4 AEM mice. Expression of only one additional gene approached statistical significance compared to the control group (IL15 was 1.5-fold up-regulated, P<0.1). Th: T helper; FLT: flight mice; AEM: animal enclosure module (control mice housed on ground).

### Cancer-related Genes in Thymus

**Figure 3:**
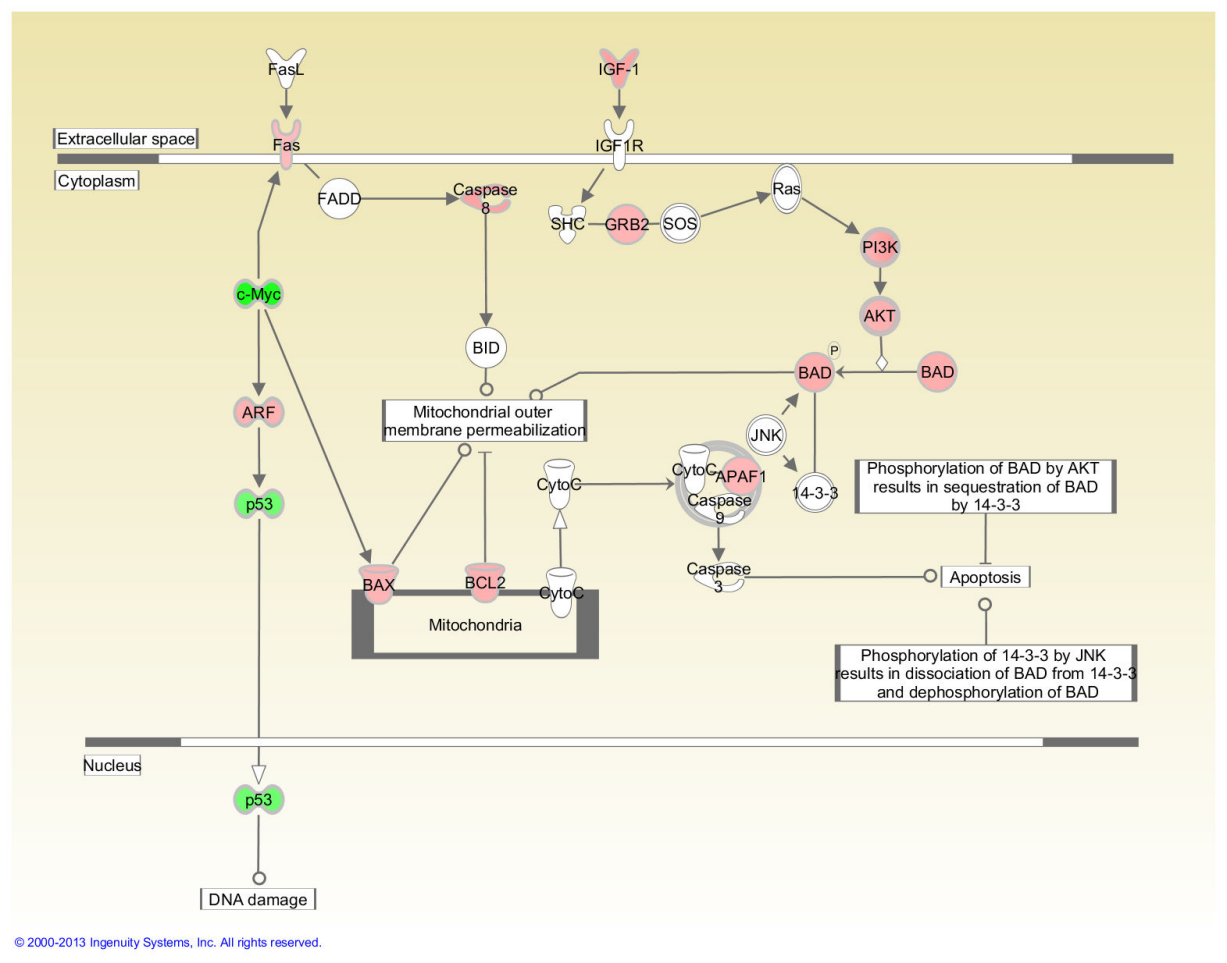
Impact of space flight on Myc-mediated apoptosis signaling in the thymus. Pathways are modified versions of the canonical pathways generated with IPA software. The up-down-regulated genes are from the cancer gene array. Red = Up-regulated. Green = Down-regulated. White = Not measured. Further description of the legend can be found at the IPA website indicated in the Materials and Methods. This analysis was based on gene expression levels in thymuses obtained from 4 AEM and 3 FLT mice.

**Figure 4:**
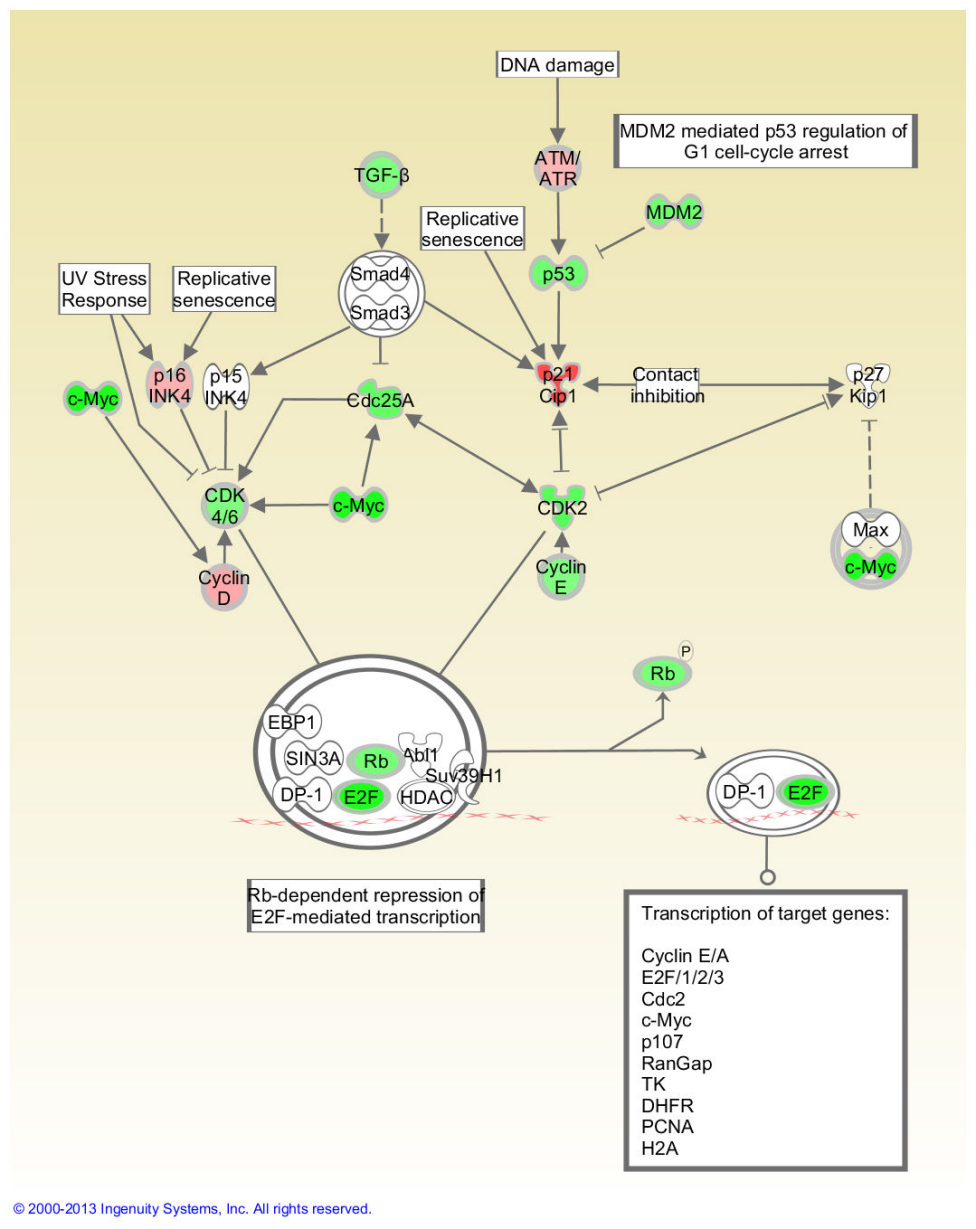
Impact of space flight on G1/S checkpoint regulation in the thymus. Pathways are modified versions of the canonical pathways generated with IPA software. The up-down-regulated genes are from the cancer gene array. Red = Up-regulated. Green = Down-regulated. White = Not measured. Further description of the legend can be found at the IPA website indicated in the Materials and Methods. This analysis was based on gene expression levels in thymuses obtained from 4 AEM and 3 FLT mice.

Table 2 shows that 15 cancer-related genes were significantly modulated in FLT thymuses compared to the AEM ground controls (P<0.05) out of a total of 84 evaluated. Of these genes, 11 were up-regulated (*Figf*, *Ncam1*, *Serpinb2*, *Itga4*, *Hgf*, *Fgfr2*, *Pik3r1*, *Sykb*, *Pdgfa*, *Casp8*, *Igf1*) and four were down-regulated (*Myc*, *E2f1*, *Mmp9*, *Cdc25a*). Fold-change was greater than 2.0 in three of these genes and all were up-regulated. Table 2 also presents the genes with a trend toward significant modulation (P = 0.05 to <0.1); nine were up- and one was down-regulated. The placement of highlighted genes in the Myc and G1/S pathways are shown in Figures 3 and 4, respectively.

**Table 2:** Fold-change in cancer-related genes in thymuses from the FLT mice vs. the AEM control group.

	**Gene**	**Fold-change**	**Description**
P<0.05	Figf	2.75	C-fos induced growth factor
	Ncam1	2.49	Neural cell adhesion molecule 1
	Serpinb2	2.47	Serine (or cysteine) peptidase inhibitor, clade B, member 2
	Itga4	1.82	Integrin alpha 4
	Hgf	1.80	Hepatocyte growth factor
	Fgfr2	1.68	Fibroblast growth factor receptor 2
	Pik3r1	1.56	Phosphatidylinositol 3-kinase, regulatory subunit, polypeptide 1
	Sykb	1.55	Spleen tyrosine kinase
	Pdgfa	1.54	Platelet derived growth factor, alpha
	Casp8	1.37	Caspase 8
	Igf1	1.39	Insulin-like growth factor 1
	Myc	-1.83	Myelocytomatosis oncogene
	E2f1	-1.82	E2F transcription factor 1
	Mmp9	-1.78	Matrix metallopeptidase 9
	Cdc25a	-1.27	Cell division cycle 25 homolog A (*S. pombe*)
P = 0.05 to <0.1	Serpine1	3.08	Serine (or cysteine) peptidase inhibitor, clade B, member 2
	Plaur	1.92	Plasminogen activator, urokinase receptor
	Itga3	1.89	Integrin alpha 3
	Col18a1	1.71	Collagen, type XVIII, alpha 1
	Itga2	1.69	Integrin alpha 2
	Cdh1	1.66	Cadherin 1
	Egfr	1.56	Epidermal growth factor receptor
	Angpt1	1.51	Angiopoietin 1
	Nfkb1	1.21	Nuclear factor of kappa light polypeptide gene enhancer in B-cells 1
	Cdk2	-1.35	Cyclin-dependent kinase 2

Data were obtained using the Mouse Cancer PathwayFinder RT^2^ Profiler^TM^ PCR Array (PAMM-033A). There were 3 FLT and 4 AEM mice. FLT: flight mice; AEM: animal enclosure module (control mice housed on ground).

### Cancer-related Genes in Spleen

**Figure 5:**
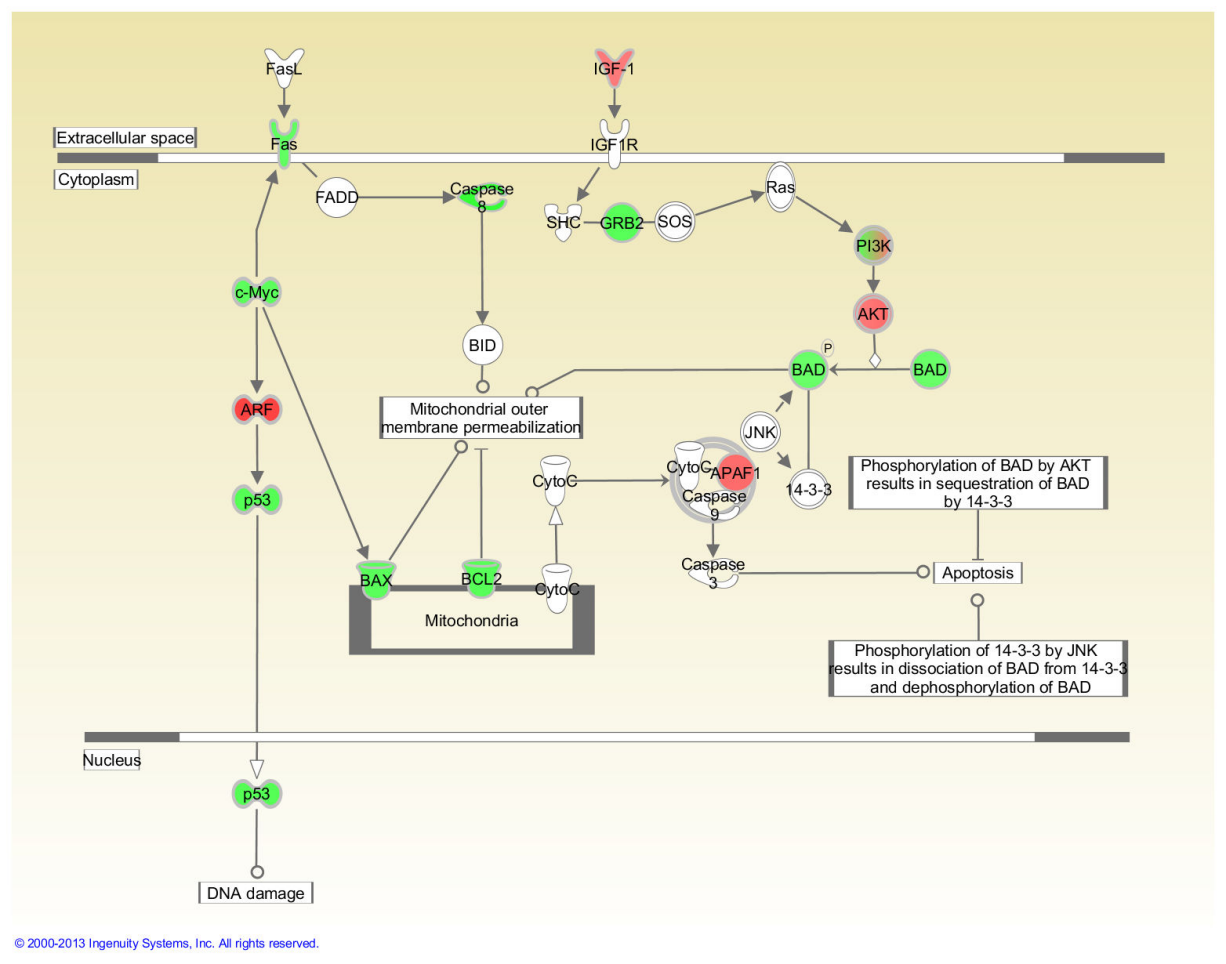
Impact of space flight on Myc-mediated apoptosis signaling in the spleen. Pathways are modified versions of the canonical pathways generated with IPA software. The up-down-regulated genes are from the cancer gene array. Red = Up-regulated. Green = Down-regulated. White = Not measured. Further description of the legend can be found at the IPA website indicated in the Materials and Methods. This analysis was based on gene expression levels in spleens obtained from 5 AEM and 4 FLT mice.

**Figure 6:**
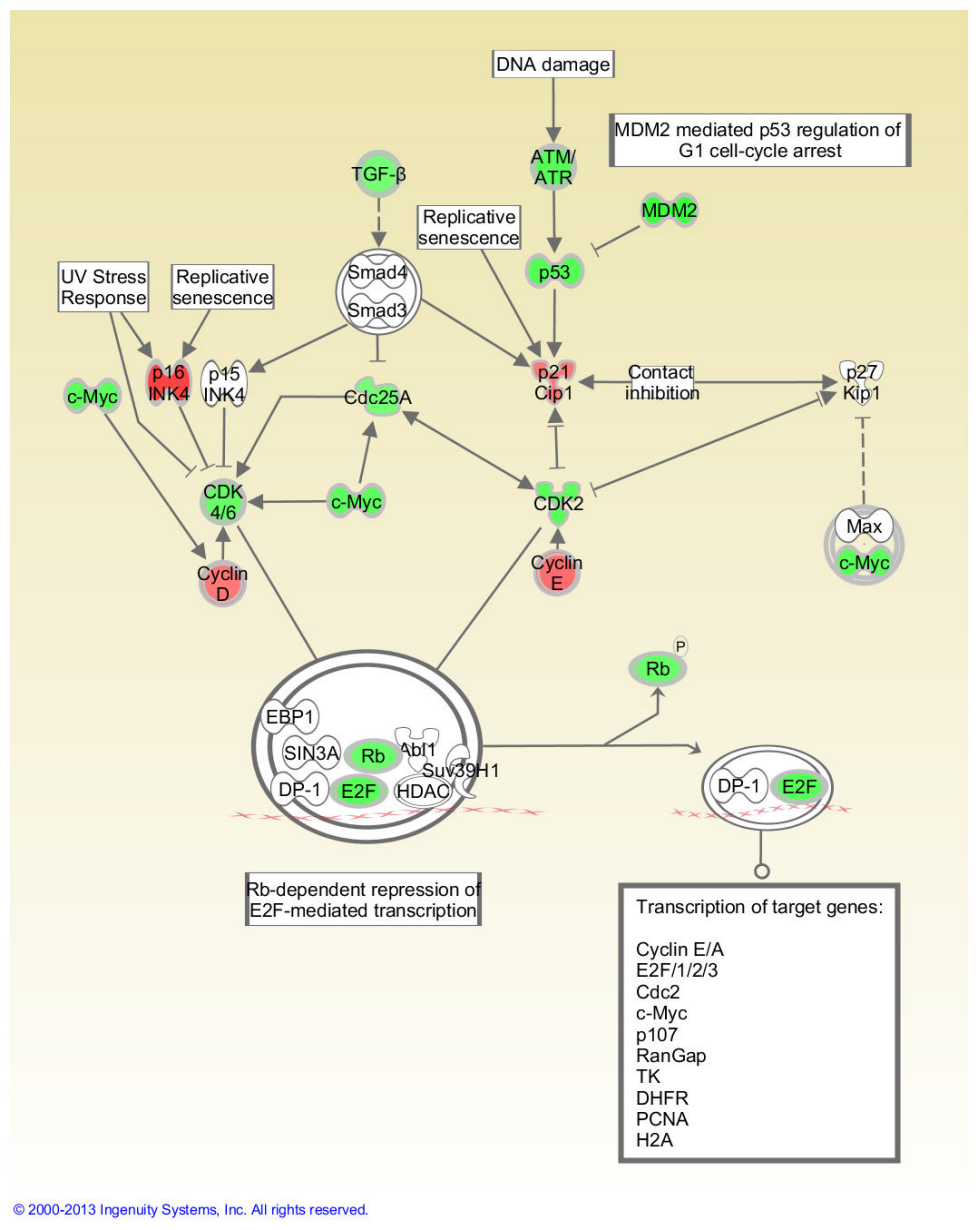
Impact of space flight on G1/S checkpoint regulation in the spleen. Pathways are modified versions of the canonical pathways generated with IPA software. The up-down-regulated genes are from the cancer gene array. Red = Up-regulated. Green = Down-regulated. White = Not measured. Further description of the legend can be found at the IPA website indicated in the Materials and Methods. This analysis was based on gene expression levels in spleens obtained from 5 AEM and 4 FLT mice.

Table 3 presents the 8/84 significantly modulated genes in spleens from FLT mice when compared to the AEM ground controls (P<0.05). Of these genes, only one was up-regulated (*Cdkn2a*), whereas seven were down-regulated (*Map2k1*, *Casp8*, *Birc5*, *Mdm2*, *Pdgfa*, *Nfkb1*, *Ctnnb1*). Also shown in Table 3 are the genes with near-significant modulation (P=0.05 to <0.1); five were up- and six were down-regulated. The placement of highlighted genes in the Myc and G1/S pathways are presented in Figures 5 and 6, respectively.

**Table 3:** Fold-change in cancer-related genes in spleens from the FLT mice vs. the AEM control group.

	**Gene**	**Fold-change**	**Description**
P<0.05	Cdkn2a	1.47	Cyclin-dependent kinase inhibitor 2A
	Map2k1	-1.67	Mitogen-activated protein kinase kinase 1
	Casp8	-1.50	Caspase 8
	Birc5	-1.48	Baculoviral IAP repeat-containing 5
	Mdm2	-1.45	Transformed mouse 3T3 cell double minute 2
	Pdgfa	-1.42	Platelet derived growth factor, alpha
	Nfkb1	-1.31	Nuclear factor of kappa light polypeptide gene enhancer in B-cells 1
	Ctnnb1	-1.26	Catenin (cadherin associated protein), beta 1
P = 0.05 to <0.1	Plau	1.62	Plasminogen activator, urokinase
	Itga3	1.48	Integrin alpha 3
	Figf	1.40	C-fos induced growth factor; Vegfd
	Mmp2	1.32	Matrix metallopeptidase 2
	Plaur	1.30	Plasminogen activator, urokinase receptor
	Tert	-1.65	Telomerase reverse transcriptase
	Itgb3	-1.31	Integrin beta 3
	Nme4	-1.28	Non-metastatic cells 4, protein expressed in
	Trp53	-1.26	Transformation related protein 53
	Grb2	-1.24	Growth factor receptor bound protein 2
	Bax	-1.23	Bcl2-associated X protein

Data were obtained using the Mouse Cancer PathwayFinder RT^2^ Profiler^TM^ PCR Array (PAMM-033A). There were 4 FLT and 5 AEM mice. FLT: flight mice; AEM: animal enclosure module (control mice housed on ground).

## Discussion

The data show that there was some degree of atrophy in both organs collected from the FLT mice shortly after landing compared to AEM controls, although statistical significance was obtained only for the spleen. Overall, this is consistent with our previous findings on rodents flown in space and is likely due to a combination of stressors that lead to loss of sensitive cells in these body sites. We have previously found decreases in leukocyte populations in rodent models after three space shuttle flights (STS-77, STS-108 and STS-118) that had duration times similar to the STS-135 flight [20,37,40,41]. A possible contributing factor is the relatively low volume of water consumed by the FLT mice, i.e., approximately 19% less than the amount consumed by the AEM controls. Hyperosmolarity, which occurs during dehydration, has been reported to decrease cellularity in spleens and thymuses of mice [45]. However, the degree of dehydration did not appear to be severe; personnel inspecting the animals after landing found no obvious signs of ill health.

Evaluation of thymus samples using the TUNEL assay revealed that the FLT group had significantly more DNA fragmentation compared to the AEM group. Damage to DNA has long been associated with apoptotic cell death. Research indicates, however, that necrosis, autophagy and mitotic catastrophe are also possibilities [46,47]. A decrease in leukocytes, regardless of specific mechanism, could certainly contribute to low mass of immune-related organs. It seems likely that the increased DNA fragmentation in FLT thymuses includes the combination of microgravity and increased radiation while in space plus the hypergravity experienced during take-off and landing (maximum of approximately 3-G). A study of brains from rats kept under simulated weightlessness followed by hypergravity found that the highest level of apoptotic cells based on TUNEL-positivity occurred in animals subjected to both conditions [48]. In addition, studies with cells (including lymphocytes) subjected to simulated microgravity on Earth have demonstrated numerous abnormalities including mitochondrial disruption and apoptosis [49].

Because we were part of NASA’s large BSP-organized team, the tissue fixation process for the TUNEL assay was not ideal. In terms of section thickness, 6 µm was chosen as the standard for all organ samples scheduled for histology, when slices <4 µm would have been better for this particular assay. The thymus has high cell density, thus making it a challenge for us to count TUNEL-positive cells based on total cell nuclei. In order to normalize the quantification process and minimize stochastic error, we therefore adapted our quantification methods and counting criteria using ImageJ software on digital microphotographs. Cell density profiles were expressed as mean number of positive cells per square millimeter for the TUNEL assay. Similar procedures have been described in the literature for various tissues that include the thymus [43,44,50].

Our results do show increased DNA fragmentation in the thymuses from FLT mice, thus supporting our hypothesis. This information increases the importance to study immune/cellular mechanisms triggered by stress stimuli, especially in the context of space flight.

The T cell-related genes evaluated in the thymus included those associated with Th1 and Th2 cytokines, CD4^+^ T cell markers, the T regulatory (Treg) cell network, T and B cell activation, relevant transcription factors and transcription regulators. The data showed that the expression of six genes was significantly different in the FLT group compared to the AEM group. The fact that relatively few of these genes displayed significant modulation may be at least partly due to the relative immaturity of thymocytes compared to Th cells in the periphery. Another contributing factor may be related to thymic atrophy in the FLT mice which likely triggered regenerative mechanisms that may have further increased the proportion of immature cells. The most up-regulated gene was *Il10* that encodes interleukin-10 (IL-10), a cytokine produced by several leukocyte types including T lymphocytes. IL-10 is a potent immunosuppressor that is crucial for limitation and ultimate termination of inflammatory responses [51]. However, it is a pleiotropic cytokine that can also enhance B cell survival, antibody production and phagocyte function [52]. In our previous study of STS-118 mice, IL-10 level was high in supernatants of spleen cells from the FLT animals after activation with anti-CD3 monoclonal antibody [37], indicating that the capacity to produce this cytokine was enhanced after landing. In blood from astronauts who experienced reactivation of latent herpes virus infection during short-duration spaceflights, elevated IL-10 has been reported shortly after landing [53].

Genes encoding IL-18 receptor 1 (*Il18r1*) and IL-18 binding protein (*Il18bp*) were also up-regulated in FLT thymuses compared to the AEM group. IL-18, together with several other cytokines, induces phenotypic and functional changes in immature cells within the thymus that can facilitate T cell development [54]. Expression of the *Il18* gene itself in the FLT and AEM thymuses, however, was similar at the time of assessment. The other up-regulated gene was *Spp1* that encodes secreted phosphoprotein 1 (perhaps better known as osteopontin, OPN). OPN is a multifunctional protein involved in adhesion, signaling and survival of cells, as well as tissue remodeling and immune regulation [55,56]. Its level is increased in response to various stressors [57]. In a space flight simulation with hind limb unloaded mice (wild type and OPN knockouts), it was demonstrated that OPN was required for thymus and spleen atrophy and loss in body mass [58]. Hence, it seems possible that enhanced OPN production (up-regulated *Spp1*) may have been involved in the relatively low thymus mass noted in the present study.

*Il6*, encoding IL-6, was one of the two down-regulated genes in FLT compared to AEM thymuses. Since this cytokine is known to cause thymic involution [59], the decreased expression of *Il6* in our FLT mice is consistent with the need for regeneration. Furthermore, since IL-6 stimulates immune responses when needed, e.g. during infection [60], susceptibility to microbes may be increased when *Il6* is down-regulated. However, after a 7-day mission in space (STS-54), mitogen-induced production of IL-6 by rat thymocytes was enhanced compared to control rats housed on ground [61]. *Ccl7* was the other down-regulated gene in the FLT versus AEM comparison. This gene encodes chemokine (C-C motif) ligand 7 (CCL7; originally called monocyte-specific chemokine 3, MCP-3), a small protein that attracts monocytes and regulates macrophage function [62]. Thus, the decreased expression of *Ccl7* in the present study suggests an anti-inflammatory response in FLT thymuses.

Status of genes relevant to cancer was determined in both thymus and spleen. The array included genes important in cell cycle control, DNA damage repair, apoptosis, cell senescence, signal transduction, transcription, adhesion, angiogenesis, invasion and metastasis. Of the genes we assessed, more were affected in the thymus than in the spleen when the FLT group was compared with the AEM controls. Of the 84 genes evaluated in each organ, the expression of only *Figf*, *Ncam1* and *Serpinb2* was modulated by more than 2-fold. All three of these genes were in the thymus and all three were up-regulated. Of these genes, the expression of *Figf*, encoding c-fos induced growth factor, was the most enhanced. This protein belongs in the platelet-derived growth factor/vascular endothelial growth factor (PDGF/VEGF) family and is also known as VEGF-D. It promotes angiogenesis and endothelial cell growth, thus facilitating tumor progression [63,64]. Enhanced expression of *Figf* was also evident in our previous study of mouse thymuses after return from the STS-118 flight [37]. *Ncam1* encodes neural cell adhesion molecule (NCAM, often referred to as cluster of differentiation 56 or CD56). Expression of CD56 on a variety of malignant and pre-malignant cells has been reported in patients, including those with precursor T-cell lymphoblastic leukemia/lymphoma (T-ALL/LBL) cells [65-68]. The *Serpinb2* (serine peptidase inhibitor, clade B member 2; also known as plasminogen activator inhibitor 2 or PAI-2) is a member of the large serpin family. Although the functions of this molecule are not entirely clear, there is evidence that it may decrease risk for tumor metastasis, inhibit apoptosis and protect against cytotoxic effects of viruses [69-71]. *Casp8* and *Pdgfa* were the only genes significantly affected in both the thymus and spleen. However, these two genes were up-regulated in the thymus, but down-regulated in the spleen, indicating that body compartment can make a great difference in the results.

In our previous study using the same cancer gene array on thymus samples from mice flown on STS-118, a very different profile was obtained [37]. The number of cancer-related genes affected in the thymus from FLT mice in the present study (15/84) was less than what we found in FLT mice flown on STS-118 (30/84). Previously, there were four up- and nine down-regulated genes in the FLT group versus the AEM group, but only the enhanced expression of *Figf* was noted after both flights. The most likely explanation for this discrepancy is that the STS-118 mice were subjected to muscle strength testing and nuclear magnetic resonance body composition measurements by investigators prior to euthanasia and collection of samples for further testing at our institution. The extent of these procedures, and the resulting stress generated by involving the mice in these assays, on gene expression profiles is not known. Possible additional contributing factors to the discrepancy between the two flights are differences in the method of euthanasia and the commercial source of the mice [72]. The impact of these latter factors, if present, seems likely to be minor. We believe that the results from the present study more accurately reflect the modulatory effects of stressors associated with space flight, since mice flown on STS-118 underwent additional procedures (as noted above) prior to sacrifice and organ collection.

The cancer gene expression profiles were, indeed, quite different for the thymus and spleen. However, this is not entirely surprising, since gene expression tends to reflect organ-specific cell composition and function at the molecular level. Organ variability in gene expression has been previously reported in animal models, as well as in humans [73,74]. Furthermore, lack of correlation in gene regulation between tissue types is common under microgravity conditions. In fact, most studies have described anomalies in the typical gene expression patterns in well-known pathways. For example, Singh and colleagues have detected epigenetic changes under microgravity conditions [75], thereby suggesting that these changes may be the result of alternative folding/conformation state of the DNA which then can give rise to alternative gene expression.

To determine the effects of space flight on relevant processes, we relaxed the statistical constraints to focus on fold changes and limited the analysis to cancer-related genes in two specific canonical pathways: Myc Mediated Apoptosis Signaling and Cell Cycle: G1/S Checkpoint Regulation. The importance of these pathways in carcinogenesis has been well documented [76,77]. Although pathway analysis works best with large numbers of genes and we included only the cancer-related genes, we believe this analysis is justified for three reasons: 1) the generated data are unique; 2) the availability of the shared tissues was very limited and repetition is unlikely in the near future as this was the last flight of NASA’s Space Shuttle Program; and 3) the sample size for this study was somewhat limited, particularly as transformation to an abnormal phenotype and immune function were not primary goals for this space shuttle experiment.

In the pathway analysis*, Myc* and *p53* appear to be down-regulated in both the thymus and spleen suggesting that DNA damage-induced apoptosis is not a major factor after space flight (Figures 3 and 5). Similarly, mitochondria-dependent apoptosis also appears to be down-regulated in both organs. In the spleen, this is indicated by the down-regulation of caspase 8 and *BAX*, both known to induce mitochondrial membrane permeabilization. While these genes are actually up-regulated in the thymus, along with a similar increase in the expression of *BAD*, their impact on membrane permeability are minimized by a corresponding up-regulation of *BCL2* in the mitochondria and the activation of *IFG-1/Ras* pathways in the cytoplasm. The latter results in the activation of *AKT* and the subsequent phosphorylation of both *BAD* and caspase 8. When phosphorylated, *BAD* cannot bind to *BCL2* and caspase 8 cannot be activated, thereby minimizing mitochondrial-dependent apoptosis. Although there is a similar up-regulation of *IGF-1* and *AKT* in the spleen, the down-regulation of BAX in the mitochondria makes a full activation of the *IFG-1*/*Ras* pathway unnecessary.

Surprisingly, despite the down-regulation of apoptosis signaling and space flight-induced decreases in virtually all immune populations (often reported previously), cell cycle progression appears to be arrested in both the spleen and thymus based on pathway analysis (Figures 4 and 6). This is indicated by a space flight-induced down-regulation of virtually all G1/S checkpoint genes (including *Cdc25A*, *CDK2, CDK4/6, Rb and E2F*) with a corresponding up-regulation of cell cycle progression inhibitors (including both *p16INK4* and *p21/Cip1*). This suggests that immunocyte recovery, if occurring at all immediately after flight, is not a primary function of the thymus or spleen.

Because *p21* is associated also with cell differentiation, the up-regulation of this gene in the thymus, combined with the apparent cell cycle arrest, suggests that T cell maturation may be enhanced. Although the expression of *Il18* was not affected, the increased expression of *IL18bp* and *IL18r1* tends to support this possibility [54,78]. However, genes encoding cytokines known to be involved in thymic regeneration, e.g., *Il7*, *Il15*, *Il17*, *Il22* and *Il23* [78-81] were not significantly up-regulated by space flight. Indeed, the cytokine-related genes that were altered in the thymus (*Ccl7*, *Il6*, *Il10 and Il18bp*) are all involved in the down-regulation of cell-mediated immunity, including T cell differentiation. Similarly, with the exception of *Il18r*, there were no significant space flight-induced changes in many of the surface receptor genes commonly associated with Th cell differentiation that were included in the array (*Cd28*, *Cd40*, *Cd40lg*, *Cd80*, *Cd86*, *IL2Rα*, *Il4ra* and *Il12rb2*). A previous study of granulocytic lineage cells in the bone marrow from mice shortly after Space Shuttle Endeavour (STS-118) landed found a higher level of differentiated and lower level of less differentiated cells compared to AEM-housed ground controls, and there was no radical disruption in the distribution of subpopulations [39].

In conclusion, the data clearly show that shortly after a 12.8 or ~13-day flight in space there was a significant impact on the assessed parameters. Spleen mass was especially reduced, increased DNA fragmentation was noted in the thymus and a number of important genes associated with T cells and cancer were modulated by flight compared to the AEM ground controls. Although gene expression in FLT mice was not always greater than 2-fold different compared to the AEM controls, we believe it is important to present all significantly modified genes due to the uniqueness of the study and because there is no compelling evidence that a greater than 2-fold change always has a greater biological impact than a less than 2-fold change. In future space flight studies, it would be important to evaluate protein levels associated with these genes. Pathway analysis suggested down-regulation of apoptosis and inhibition of cell cycling. Since the FLT group had an abundance of apoptotic/dead cells, the down-regulation of apoptosis signaling and cell cycling pathways in the remaining viable cells (those still capable of gene expression) may reflect a great need for survival. Multiple factors could certainly be responsible for the changes, including altered gravity, increased radiation and stress of handling, as well as the body’s attempt to regain homeostasis after return to Earth. It remains to be determined whether the aberrations are persistent and translate into increased risk for pathologies such as leukemia/lymphoma. These malignancies have been identified as a dominant risk to astronauts exposed to an SPE (as indicated at the NASA Technical Reports Server). Finally, in September 2011, NASA announced that it will proceed with a new Space Launch System that will allow human travel into space further than ever before. Thus, a better understanding of the precise mechanisms that result in immunological changes and possible health consequences associated with space travel should remain a high research priority so that appropriate countermeasures can be developed.
