# Correction: Changes in Mouse Thymus and Spleen after Return from the STS-135 Mission in Space

**DOI:** 10.1371/annotation/e66bdc4e-2409-4582-b163-7bc182db275e

**Published:** 2013-09-27

**Authors:** Daila S. Gridley, Xiao Wen Mao, Louis S. Stodieck, Virginia L. Ferguson, Ted A. Bateman, Maria Moldovan, Christopher E. Cunningham, Tamako A. Jones, Jerry M. Slater, Michael J. Pecaut

There was an error in the Funding statement. The correct version of the Funding statement is available below.

The work was supported by NASA grant NNX10AJ31G and the LLUMC Department of Radiation Medicine. The funders had no role in study design, data collection and analysis, decision to publish, or preparation of the manuscript. 

